# Ultrasound-guided femoral approach for coronary angiography and interventions in the porcine model

**DOI:** 10.1038/s41598-022-17436-0

**Published:** 2022-08-17

**Authors:** Grigorios Tsigkas, Georgios Vasilagkos, Alexandros Tousis, Michail Theofanis, Anastasios Apostolos, Ioannis Spyridonidis, Leonidas Goudas, Georgios Karpetas, Athanasios Moulias, Christos S. Katsouras, Panagiotis Kitrou, Virginia Mplani, Anargyros N. Moulas, Dimitrios Karnabatidis, Periklis Davlouros

**Affiliations:** 1 Department of Cardiology, School of Medicine, University of Patras, Patras, Greece; 2 Department of Radiology, School of Medicine, University of Patras, Patras, Greece; 3 Department of Anesthesiology, School of Medicine, University of Patras, Patras, Greece; 4 Department of Cardiology, School of Medicine, University of Ioannina, Ioannina, Greece; 5 General Sciences Department, University of Thessaly, Volos, Greece

**Keywords:** Cardiovascular models, Interventional cardiology

## Abstract

Coronary angiography and percutaneous coronary intervention (PCI) procedural details in swine are similar to those performed to humans, since their heart and coronary anatomy closely resembles. However, only a few detailed descriptions of the procedure are available, containing notable differences. We present a feasible and reproducible protocol for percutaneous coronary interventions in porcine experimental models, utilizing ultrasound-guided femoral approach. Nine female pigs were studied to explore the feasibility of superficial femoral arterial (SFA) access for coronary angiography and provisional PCI, as well as the most suitable guiding coronary catheters and angiographic projections for the above interventions. Experiments were performed under general anesthesia, using ultrasound-guided puncture of the SFA to gain arterial access. The Amplatzer AR1^®^ catheter, and the Right Coronary Bypass^®^ catheter were used for the selective engagement of the right and the left coronary artery, respectively. Successful arterial access and subsequent cardiac catheterization were performed in all pigs. Only one animal required a second puncture for femoral artery access. None of the 9 animals presented any significant tachycardia or hypotensive episode. One animal developed an access site-related complication following the first catheterization procedure. During follow-up, 100% success of SFA catheterization was achieved using the same ultrasound-guided technique. The ultrasound-guided superficial femoral artery access for coronary angiography and provisional interventions in porcine models is a quick and safe alternative to the carotid artery approach. The RCB and AR1 catheters may be the best choice for the quick and easy selective coronary engagement of the right and left ostia, respectively.

## Introduction

Coronary procedures on animal models are widely performed to investigate novel cardiovascular therapies, prior to application on humans^1^. Since the porcine heart and coronary anatomy closely resembles that of humans, and both species have similarities regarding the innate collateral circulation, the presence of a well-developed vasa vasorum as well as a similar coagulation system, coronary angiography and percutaneous coronary intervention (PCI) procedural details are similar to those performed to humans^2–5^. As a result, porcine models are standing out as valid and safe experimental models and are widely used for the above purpose^1^.

However, only a few detailed descriptions of the procedure have been published to guide investigators performing coronary procedures. Meanwhile, notable differences in vascular access, strategies, coronary catheters used and periprocedural pharmacotherapy exist, while limited data regarding follow up interventions are available in the literature^3,6,7^.

Carotid artery access seems to be preferred as the first-line approach for experimental percutaneous procedures in porcine models, while femoral artery approach has also been described^6,8–13^. Selection of the appropriate catheters remains an unresolved issue, since coronary catheters used during those procedures in porcine models have been designed for humans.

We present our experience with ultrasound-guided femoral access for percutaneous coronary interventions in porcine experimental models, aiming to propose a structured, feasible and reproducible approach for percutaneous coronary interventions in porcine experimental models, utilizing ultrasound-guided femoral approach, examining the safety, feasibility, and reproducibility of the whole protocol.

## Methods

Nine female pigs, 5–6 months of age, weighing between 50 and 55 kg were studied to explore the feasibility of superficial femoral arterial (SFA), access for coronary angiography and provisional PCI. Additionally, we aimed to explore the most suitable guiding coronary catheters and angiographic projections for the above interventions.

### Periprocedural care

All animals were kept in individual cages, in climate-controlled rooms and were allowed a minimum of 24 h to recover from the stress of transportation before the first procedure. Food was withheld for 12 h prior to anesthesia.

All procedures performed were in accordance with the Declaration of Helsinki regarding ethical principles of medical research and were approved by the animal care and use board of Patras University Hospital’s ethics committee, and the veterinary board of Western Greece’s Provincial authorities. Furthermore, the protocol complied with the ARRIVE guidelines.

### Anesthesia and intubation

All animal experiments were performed under general anesthesia according to established anesthetic protocols^14,15^ with a combination of 15 mg/kg intramuscular ketamine (Ketamidor^®^, Richter Pharma, Wels, Austria) and 2 mg/kg xylazine (Xylan^®^, Chanelle pharmaceuticals, Loughrea, Ireland), followed by endotracheal intubation. This approach has been shown to reduce ventricular arrhythmias^6^. The intubation process has been described both on supine and prone positions with the use of a long straight-bladed Magill laryngoscope^6^.

**Figure 1:**
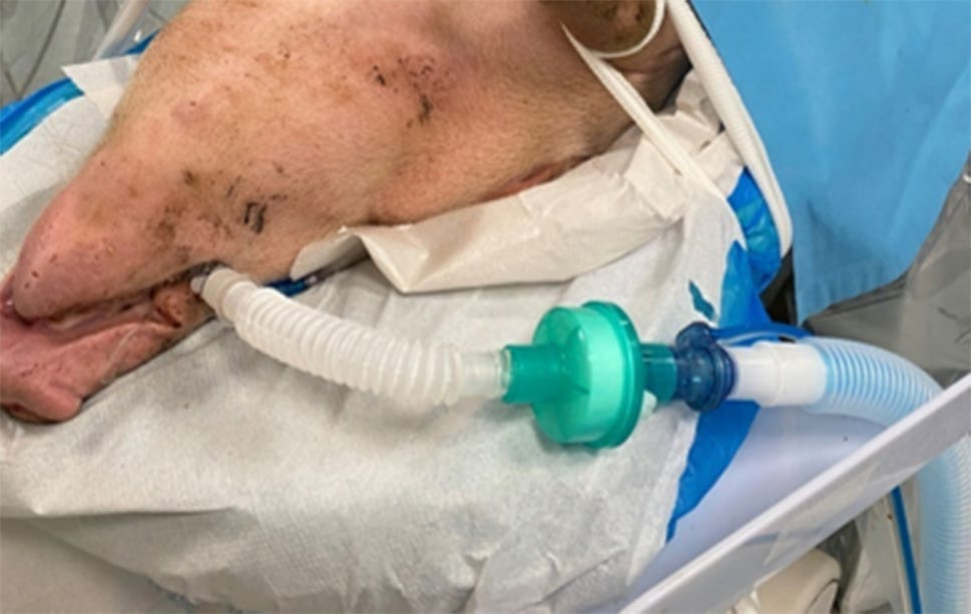
Intubated pig in supine position.

In our case, pigs were intubated in the supine position (Fig. 1), using either a standard laryngoscope or a long straight-bladed Magill laryngoscope when deemed necessary, according to the length of the oral cavity and the pharynx. An assistant held open the pig’s mouth while also applying traction to the tongue. Once the vocal cords were visualized, a standard 7 mm lubricated human endotracheal tube was inserted using a bougie. Moreover, a 20-gauge intravenous catheter was inserted to the lateral auricular vein. Both were held in place with 2–0 nylon polyamide sutures.

Gas-maintained anesthesia is generally advised for such procedures with either isoflurane or sevoflurane are considered as agents of choice^10,14,15^. Our procedures were performed by continuous propofol infusion at a rate of 1–1.4 mg/kg, which has been associated with sinus rhythm maintenance and atrioventricular node function protection without prolonging the ventricular refractoriness^16^.

We used a RAPHAEL Color Ventilator (Hamilton Medical, Bonaduz, Switzerland), to apply synchronized controlled mandatory ventilation with a minute ventilation of 6.4 L/min at 14 breaths/min and 462 ml exhaled tidal volume. Pulse oximetry and heart rate were monitored throughout the operation.

### Arterial access

The SFA was chosen for arterial access. The puncturing site was scrubbed with a povidone-iodine solution and then draped in a sterile fashion.

**Figure 2:**
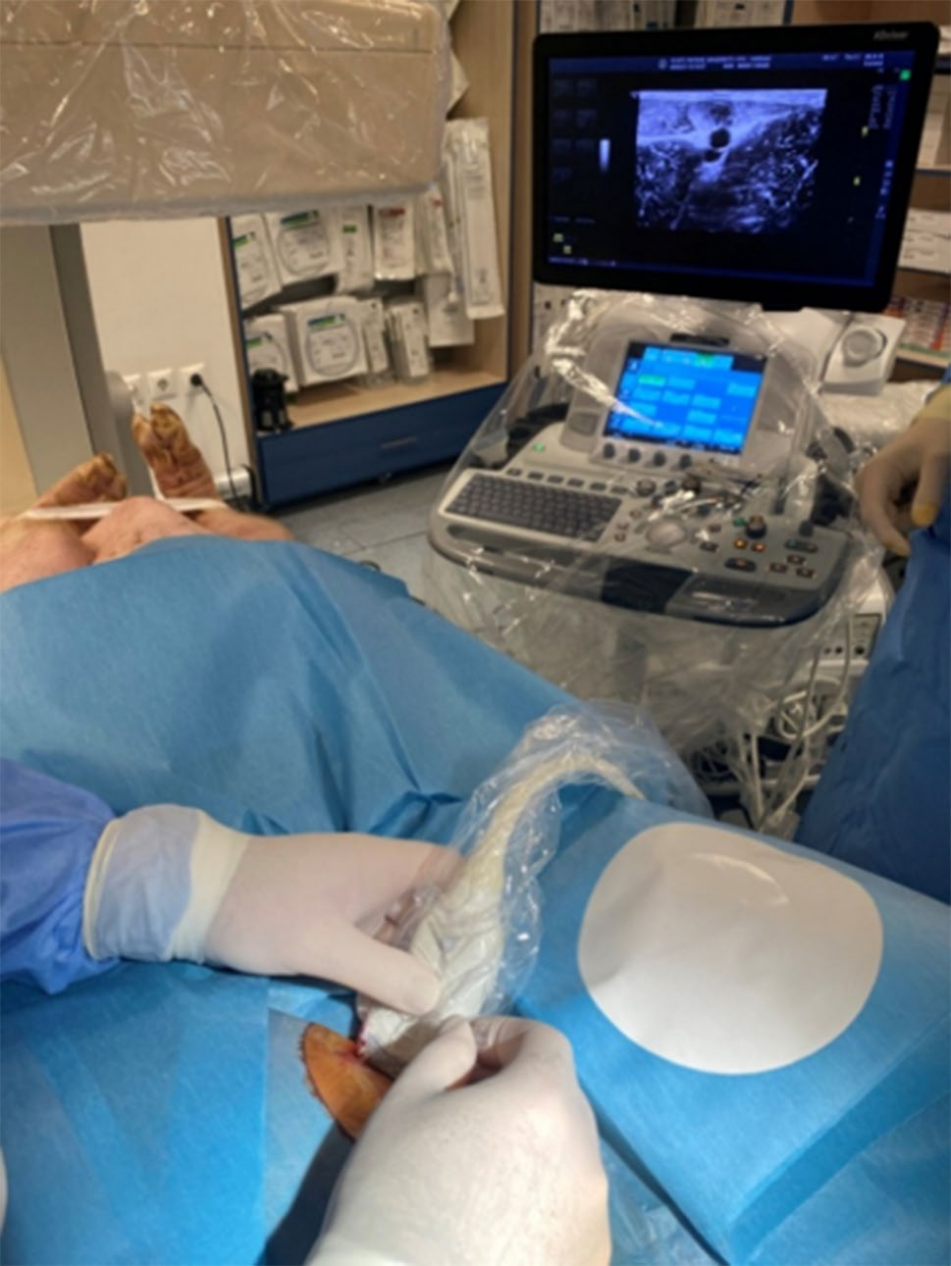
Ultrasonography-guided puncture of the right SFA.

**Figure 3:**
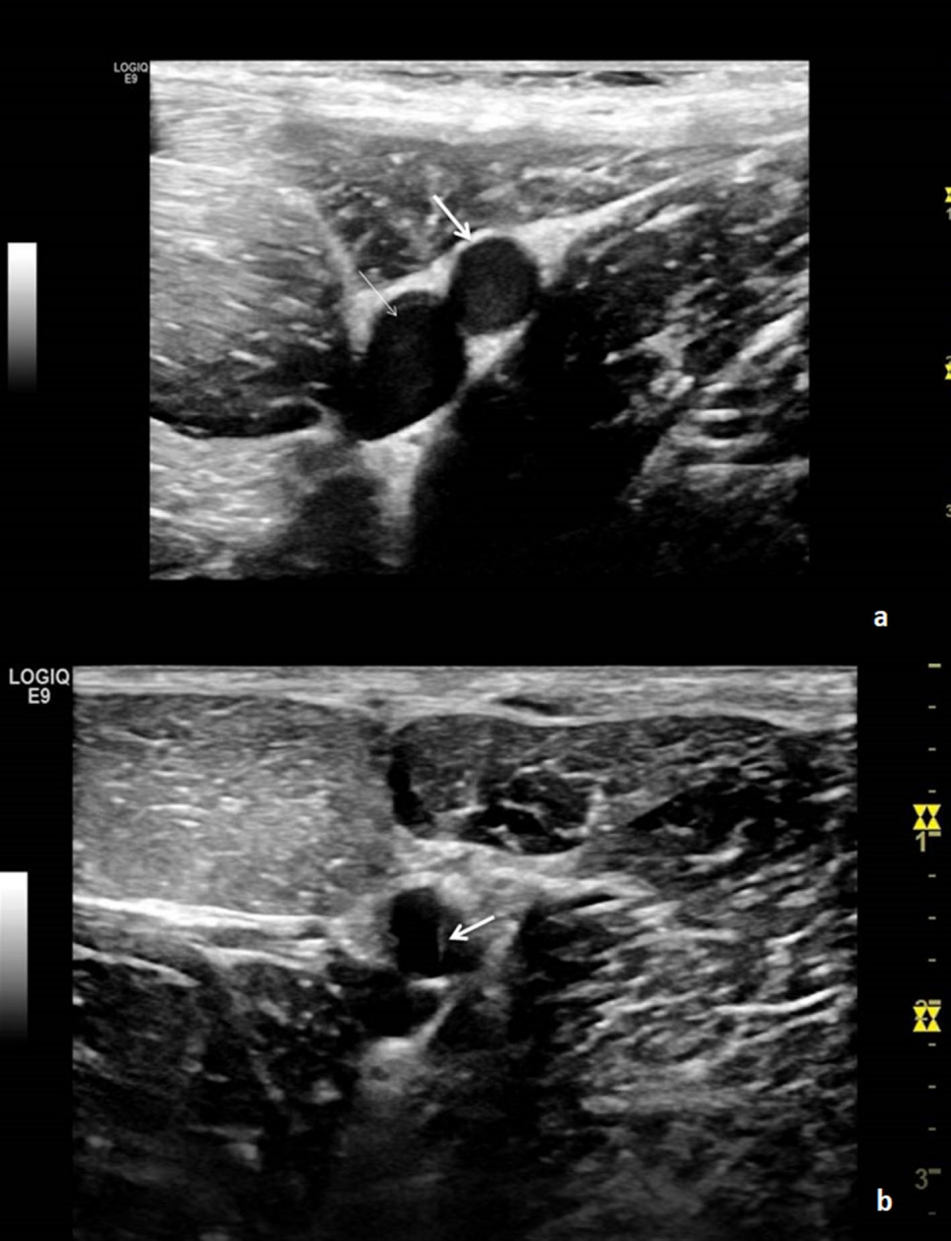
(**a**) Femoral artery (thick arrow) and vein (thin arrow), (**b**) ultrasound-guided puncture of the SFA. White arrow shows the puncture needle inserted into the SFA.

An ultrasound machine (Logiq E9—General Electric^®^, Chicago, U.S.A.) equipped with a 4–15 MHz transducer (ML6-15-D Matrix Linear Probe) was used to guide puncturing (Fig. 2). The Seldinger technique with a 21-Gauge needle was used to access the right-sided SFA^17^. After recognition of the SFA on real time ultrasound the needle was inserted at 60–80 degrees towards the artery and when pulsatile blood returned, a 0.035″ wire (Boston Scientific^®^, Marlborough, U.S.A.) was introduced (Fig. 3a,b). The needle was replaced with a 6-Fr-10 cm Radial Artery Sheath (Terumo Medical Corporation^®^, Shibuya, Japan). Once arterial access had been obtained, 100 U/kg heparin was administered to achieve systemic anticoagulation. Intra-arterial blood pressure and arterial saturation via a pulse oximeter mounted on the animal’s tail were monitored throughout the procedure. The whole procedure was carried out by three experienced operators.

### Guiding catheters

Our anecdotal experience before the current experimental study showed that for porcine models of similar age and size, the Amplatzer AR1^®^ (Cook Medical^®^, Bloomington, U.S.A.) catheter, and the Right Coronary Bypass^®^ (RCB) catheter (Cordis Corporation^®^, Hialeah, U.S.A.), might be best fitted for the selective engagement of the Right and the Left coronary artery, respectively (Table 1).

**Table 1:** Periprocedural characteristics.

N	Access-site parameters			Peri-procedural characteristics		FU-procedure parameters	
	Number of punctures (n)	Time until sheath insertion (s)	Side effects	Catheters used	Side effects	Number of punctures (n)	Time until sheath insertion (s)
1	1	60	None	AR1, RCB	Coronary artery spasm	2	60
2	1	120	None	AR1, RCB	None	3	100
3	1	57	None	AR1, RCB	None	1	30
4	1	90	Spasm, hematoma, flow absence, thrombi in FA	AR1, RCB	None	2	100
5	1	60	None	AR1, RCB	None	1	52
6	2	240	None	AR1, RCB	None	1	55
7	1	60	None	AR1, RCB	None	1	45
8	1	60	None	AR1, RCB	None	1	60
9	1	45	None	AR1, RCB	None	2	160
Mean ± SD	1.10 ± 0.33	88.00 ± 61.32				1.55 ± 0.73	73.56 ± 39.94

*FA* femoral artery, *FU* follow-up.

### Angiographic views and coronary angiography procedure

Due to differences in orientation of the heart, modifications of the standard angiographic views used in humans may be required in order to optimize visualization of the coronary arterial system in porcine models. The identification of the most suitable angiographic views has also been the objective of the current feasibility study.

**Figure 4:**
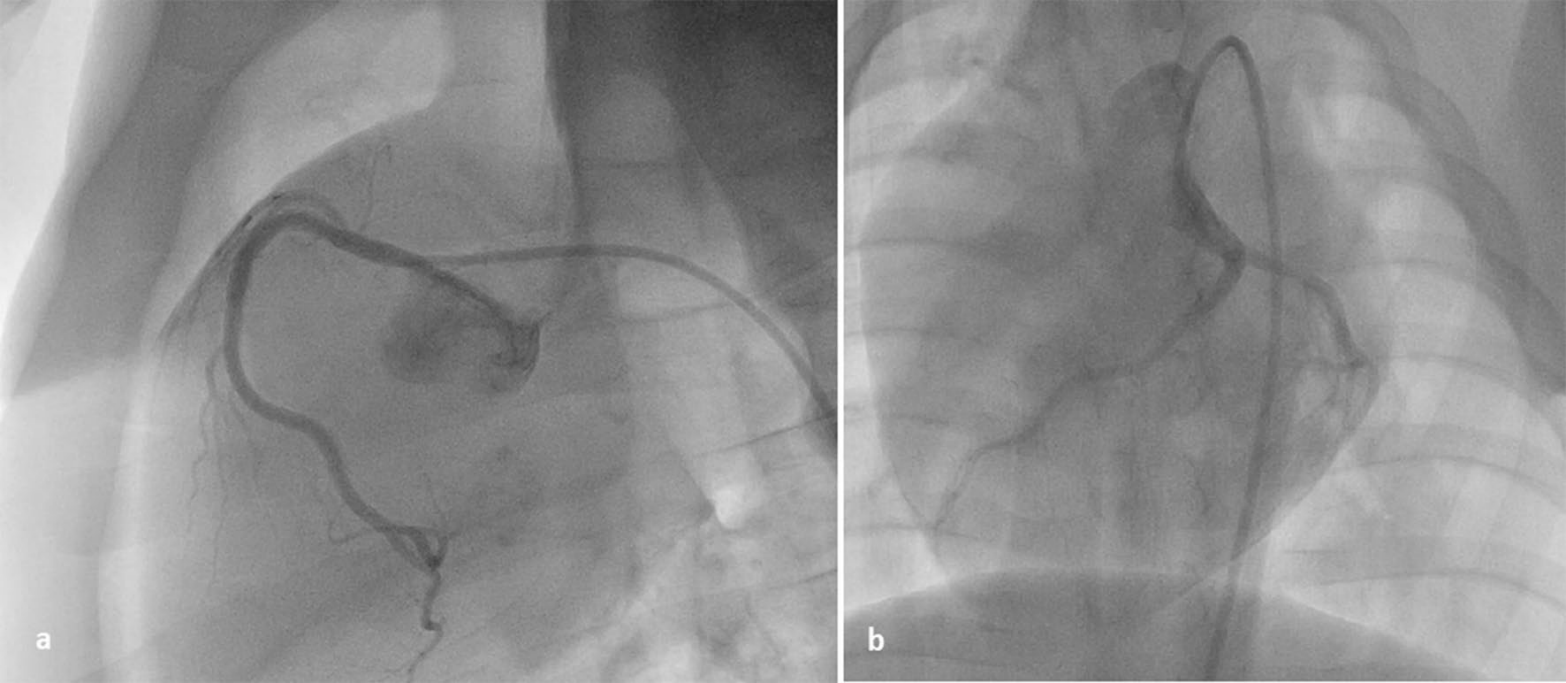
(**a**) Right coronary artery, (**b**) left main coronary artery. Notice the LAD lying to the left of the catheter’s tip.

All procedures were performed using the same angiography system (Allura Flat Panel^®^, Philips^®^, Amsterdam, Netherlands). The left anterior oblique (LAO) view was found to be optimal for the depiction of the right coronary artery and a posteroanterior cranial view for the left coronary artery (Fig. 4a,b).

### Hemostasis and recovery

**Figure 5:**
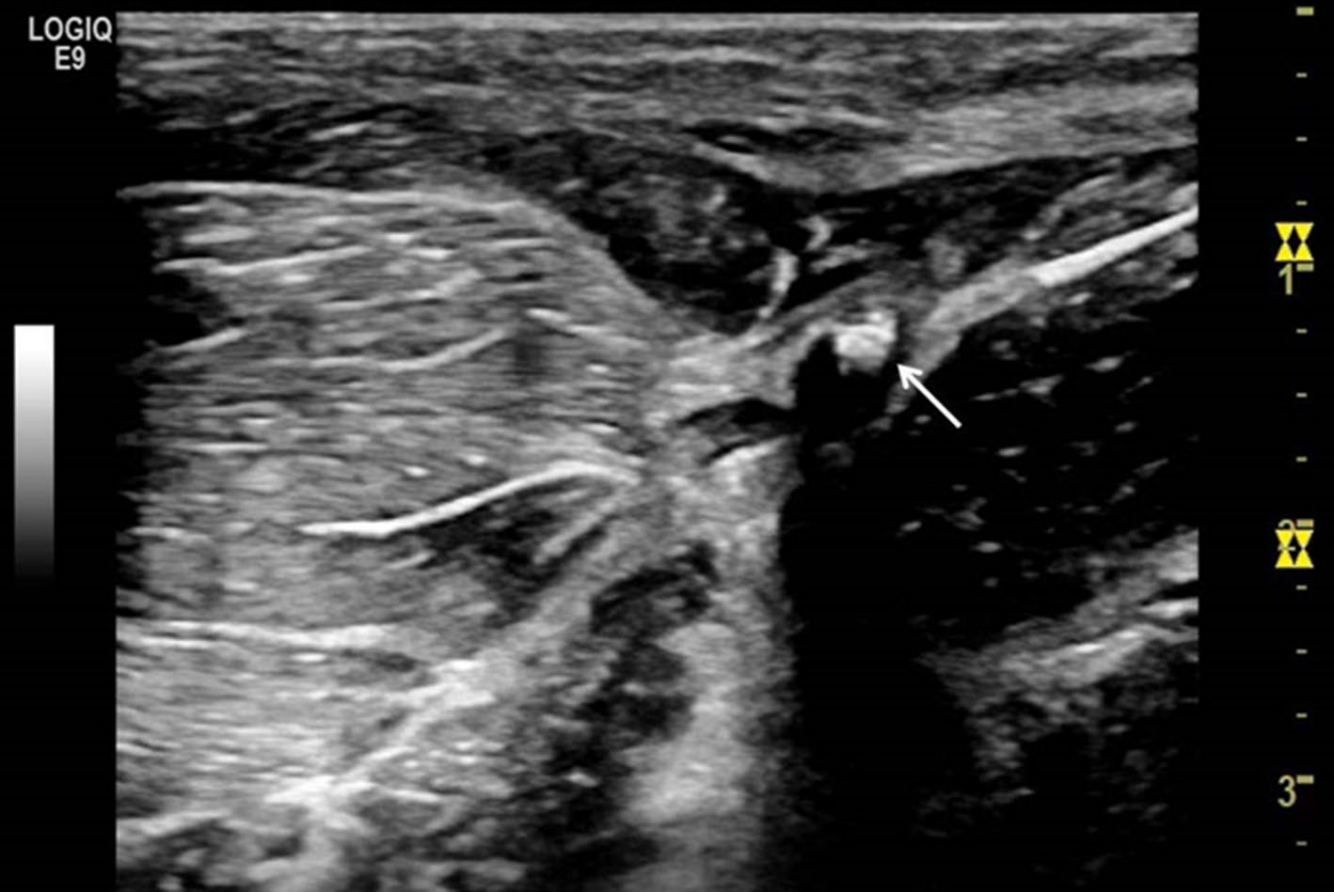
Ultrasonography of the femoral artery confirms ANGIO-SEAL^®^ VIP Vascular Closure Device placement.

After completion of the procedure, hemostasis was achieved using an arterial closure device (ANGIO-SEAL^®^ VIP Vascular Closure Device, Terumo Interventional Systems^®^, Shibuya, Japan) and the patency of the femoral artery was confirmed using Doppler ultrasonography (Fig. 5).

### Follow up and euthanasia

Thirty days after the initial procedure, reintervention using the ultrasound-guided transfemoral access was performed in the unilateral SFA. All the previously described steps were followed, and the same guiding catheters were utilized for coronary angiography.

After completion of the protocol, the animals were transferred to the recovery room and euthanasia was performed.

### Ethical approval

All applicable international, national, and/or institutional guidelines for the care and use of animals were followed.

## Results

Successful arterial access and subsequent cardiac catheterization were performed in all 9 pigs (100%). The average time required to gain right femoral access using ultrasonography-guided puncture was 88.00 ± 61.32 s. Only one animal required a second puncture for femoral artery access (mean 1.10 ± 0.33).

As described in the “Methods” section, the AR1 and RCB catheters were used, as our previous experience suggested that those catheters might be best suited for selective catheterization of porcine coronary arteries. Coronary catheterization was successful in all porcine models. Coronary artery spasm occurred in 1 animal and was relieved by intra coronary GTN administration, while none of the 9 animals presented any significant tachycardia or hypotensive episode.

One animal developed an access site-related complication following the first catheterization procedure. More specifically, the arterial ultrasound depicted a severe spasm of the SFA, a subintimal hematoma, absence of flow and a pulsing thrombus in the femoral artery, for which extra 5000 heparin units were administered. There were no clinical sequelae, and the second procedure was performed via the same artery.

During follow-up, 100% success of SFA catheterization was achieved using the same ultrasound-guided technique. The mean time for gaining arterial access was 73.56 ± 39.94 s, with a mean of 1.55 ± 0.73 punctures needed (Table 1).

## Discussion

Significant differences exist among the described strategies for coronary catheterization of porcine models^6,8^. However, a relatively high mortality rate (up to 15%), has been reported in the literature in healthy porcine models during or following coronary intervention^6^. This may in part be due to the techniques used and/or their application be relatively inexperienced operators^6^. We describe a feasible and reproducible experimental protocol for coronary angiography and provisional interventions in porcine models using an ultrasound-guided femoral access and specific guiding-catheters (AR1 and RCB).

Femoral and carotid arteries have all been proposed and studied for percutaneous arterial access in swines^9,13,18–20^. Until recently, the femoral artery was used as an access route via a surgical cut-down and sheath insertion using an arteriotomy or modified Seldinger technique. More specifically, literature suggests that the superficial femoral artery is the access-site of choice when choosing femoral artery access, partly due to its easier pulse localization and echocardiography display.

Ultrasound guidance has been used successfully in humans for arterial access, and it has been associated with less complications, punctures and procedural time^21^.

Vascular ultrasound has been applied in porcine models mainly for the carotid arterial access, and has also been tested for percutaneous needle puncture of the femoral artery for application of extracorporeal membrane oxygenation and for minimally invasive catheterization of the external jugular vein^9,12,13,22^. Recently, ultrasound guidance has been shown to facilitate femoral arterial access and reduce vascular complications in porcine models^13,14^. However, ultrasound guided femoral artery access for coronary catheterization in porcines, has not been studied extensively. According to our results the ultrasound-guided femoral artery approach was feasible, quick, easy-to-learn and safe, without causing any significant complications. Additionally, the puncture of the same femoral artery, after one month, was proven feasible and did not reveal any complications (i.e. stenosis, or obstruction) associated with the use of the vascular closure device, thus proving our proposing approach to be reproducible.

Overall, we recommend this approach as first-line choice for gaining arterial access in porcine models subjected to coronary angiography and interventions.

Despite the fact that similarities between humans’ and porcines’ heart exist, there are differences regarding the shape of the heart and its orientation in the thorax. The human heart has a trapezoidal shape, whilst the porcines’ heart, has a cone-shaped form. Additionally, the porcine heart has a rather central orientation in the thorax^23^. Therefore, classic catheter shapes, designed for coronary angiography and interventions for human patients, may fail to engage selectively the porcines’ coronary arteries. As a result, there is not any established and reproducible protocol regarding the most suitable guide catheters for experimental coronary procedure in porcines.

According to the literature, angiographic catheters used to engage the porcine coronary ostia may vary according to the arterial access used. Amplatz right and left as well as JR4 and hockey stick catheters have been used to engage both coronary arteries with the carotid artery approach^3^. JR4 was proven more suitable for the RCA while others like the standard extra backup and JL3.5 or JL4 catheters can also be useful for the LCA^6^. AR1 or hockey stick catheters can be used to engage both coronaries and JL3.5 and AL0.75 can be used to engage the LCA in the femoral approach^3,6,10^.

We found that the AR1 catheter was the best choice for the right coronary system while the RCB was best suited for the left, both allowing easy and quick selective catheterization with a 100% success rate.

The coronary tree of porcines resembles that of humans, with vessels having a similar diameter, between 2 and 4 mm^3^. The ideal projections for each coronary artery should ensure a good imaging quality, the avoidance of arterial overlapping and the ability to overview the status of the whole coronary artery with its branches. The AP and left anterior oblique (LAO) views have been described as suitable for the depiction of proximal LAD, with Cx being to the left and to the right of the guide catheter tip, respectively. An AP view as well as the RAO and LAO are useful for the RCA^6^. According to our experience, the AP cranial and the RAO cranial view is best suited for the left coronary system, whereas the LAO for the RCA.

During our protocol, only one animal suffered a transient spasm of the RCA, but that was quickly resolved with intraarterial nitrates.

## Conclusion

The ultrasound-guided superficial femoral artery access for coronary angiography and provisional interventions in porcine models is a quick and safe alternative to the carotid artery approach. The RCB and AR1 catheters may be the best choice for the quick and easy selective coronary engagement of the right and left ostia, respectively.

## Data Availability

All data generated or analyzed during this study are included in this published article. The corresponding author can be contacted if there is a request for the data.
